# Changes in the seroprevalence and risk factors between the first and second waves of COVID-19 in a metropolis in the Brazilian Amazon

**DOI:** 10.3389/fcimb.2022.932563

**Published:** 2022-09-16

**Authors:** Maria Karoliny da Silva Torres, Felipe Teixeira Lopes, Aline Cecy Rocha de Lima, Carlos Neandro Cordeiro Lima, Wandrey Roberto dos Santos Brito, Janete Silvana S. Gonçalves, Onayane dos Santos Oliveira, Vanessa de Oliveira Freitas, Bernardo Cintra dos Santos, Renata Santos de Sousa, Jayanne Lilian Carvalho Gomes, Bruno José Sarmento Botelho, Ana Carolina Alves Correa, Luiz Fernando A. Machado, Rosimar Neris Martins Feitosa, Sandra Souza Lima, Izaura Maria Vieira Cayres Vallinoto, Antonio Carlos R. Vallinoto

**Affiliations:** ^1^ Laboratory of Virology, Institute of Biological Sciences, Federal University of Pará, Belém, Brazil; ^2^ Graduate Program in Biology of Infectious and Parasitic Agents, Federal University of Pará, Belém, Brazil

**Keywords:** SARS-CoV-2, COVID-19, seroepidemiology, Belém, Amazon, Brazil

## Abstract

In Brazil, the coronavirus disease 2019 (COVID-19) epidemic spread rapidly in a heterogeneous way, mainly due to the different socioeconomic and behavioral characteristics of different regional populations and different evaluation periods. We performed a cross-sectional study including 1,337 individuals (first wave = 736/second wave = 601) after the first two waves of COVID-19 in the city of Belém, the capital of the state of Pará. The detection of IgG anti-SARS-CoV-2 antibodies was performed using an enzyme-linked immunosorbent assay test followed by statistical analysis using the RStudio program. Our results showed an increase in the seroprevalence (first wave= 39.1%/second wave= 50.1%) of anti-severe acute respiratory syndrome coronavirus 2 (SARS-CoV-2) IgG antibodies in the population of Belém from the first to the second pandemic wave. Advanced age, primary or secondary education level, lack of social isolation, and a low frequency of protective mask use were considered risk factors for SARS-CoV-2 infection during the first wave compared to the second wave. This study is one of the firsts to provide important information about the dynamics of virus circulation and the groups vulnerable to exposure in the two major periods. Our data emphasize the socioeconomic characteristics of the affected population and that nonpharmacological prevention measures are crucial for combating the pandemic.

## Introduction

As of March 19, 2022, Brazil had reported approximately 29,882,397 confirmed cases of severe acute respiratory syndrome coronavirus 2 (SARS-CoV-2) infection and approximately 659,241 deaths due to coronavirus disease 2019 (COVID-19) (Brasil, 2022a). In the state of Pará, northern Brazil, the number of cases and deaths, respectively, was higher at the peak of the first wave (from May 2020 to February 2021) of COVID-19 than at the peak of the second wave (in March 2021). The city of Belém, the capital of the state, thus far has reported 135,542 confirmed cases and approximately 5,294 deaths, with a case fatality rate (3.91%) that is higher than that observed for the state of Pará (2.42%) (SESPA, 2022). Incidence, prevalence, and mortality rates have varied throughout the country, depending on the region and the strategies adopted to control viral spread, which have been inconsistent among states of the federation over time (Ribeiro et al., 2020).

At the population level, the incidence of infection during each period, the factors associated with symptomatic or asymptomatic disease, and the duration of the antibody response against infection remain unclear. Seroprevalence studies allow a better understanding of the course of the disease in the general population and the identification of population groups less protected by public health measures, generating important information that stimulates the formulation of more efficient prevention strategies for future epidemic waves.

Therefore, our study sought to assess the seroprevalence of IgG anti-SARS-CoV-2 antibodies in the population of the city of Belém at two key moments in the COVID-19 pandemic, after the first and second wave of the pandemic, respectively. In addition, we sought to identify and compare the socioeconomic and behavioral profiles and risk factors for exposure to SARS-CoV-2 infection in the two periods to contribute to a better description and understanding of the impact of this pandemic in the city of Belém.

## Materials and methods

### Ethical aspects

The project was submitted to and approved by the National Research Ethics Committee (CONEP) and the Ethics Committee in Research with Human Beings of the Institute of Health Sciences of the Federal University of Pará (CAAE: 31800720.1.0000.0018) in compliance with the guidelines and standards of regulatory agencies for research involving human subjects. The participants were recruited from public calls published in different locations in the city of Belém (schools, churches, community centers, condominiums, and university) where health actions were carried out. Only voluntary individuals were included in the survey. Individuals already vaccinated with one or two doses against SARS-CoV-2, those who did not respond to the epidemiological questionnaire, those who did not sign the informed consent form, and those under 7 years of age were excluded from the research. There was no overlap of the individuals analyzed in the two study periods.

After signing the free and informed consent form, the individuals were interviewed using a structured questionnaire that included questions about clinical, demographic, and behavioral characteristics related to possible risk factors for SARS-CoV-2 infection. We obtained signed informed consent forms from individuals aged 18 years or older. Children aged 7–11 years and adolescents aged 12–17 years signed a free and informed assent term, and their respective parents or guardians signed an informed consent form.

### Collection, processing, and storage of samples

After completing the questionnaire and signing the informed consent form, blood samples (10 ml) were collected *via* venipuncture in a vacuum tube containing EDTA. Subsequently, the samples were processed anonymously in the virology laboratory, separating plasma and leukocytes, which were stored at -70°C.

### Study design and sampling

This cross-sectional study included 1,337 volunteer individuals residing in the city of Belém during two major periods: after the first wave of COVID-19 (October 2020 to February 2021, 736 individuals) and after the second wave of COVID-19 (March 2021 to December 2021, 601 individuals).

The following information was collected by self-declaration from each participant: (i) sociodemographic data (age, sex, income based on minimum wage, skin color), (ii) presence of comorbidities, and (iii) behavioral information about prevention measures, such as mask use, travel, hand hygiene, social distancing, and having had contact with people infected with SARS-COV-2. Considering that the current population of the city of Belém was composed of a mixture of three ethnicities (white European, indigenous, and black African) and that the three ethnic components are completely integrated in the current population, we classified the volunteers of the present study based on self-identification in relation to skin color, using the same criteria used by the IBGE (Brazilian Institute of Geography and Statistics— https://www.ibge.gov.br) to carry out the Brazilian census. Participant data were recorded using EPI Info software version TM 7.2.4 (CDC, 2021) and stored on the local server of the Virus Laboratory of the Federal University of Pará.

### Detection of anti-SARS-CoV-2 IgG antibodies

The detection of anti-SARS-CoV-2 IgG antibodies was performed using an enzyme-linked immunosorbent assay (Euroimmun, Lübeck, Germany) that utilized the recombinant structural peptide S1 (RBD domain) of the spike protein as an antigen. The protocol followed the manufacturer’s recommendations. The samples were classified as non-reagent (ratio <0.8), indeterminate (0.8 ≤ ratio ≤ 1.1) or reagent (ratio >1.1) for IgG, as suggested by the manufacturer. The assay has a clinical sensitivity of 75−93.8% (>10−20 days to ≥21 days after disease onset) and a specificity of 99.6% for IgG antibodies, according to the manufacturer’s guidelines.

### Statistical analysis

All analyses were performed in R Studio version 4.1.1 using the R packages. Associations between the presence of anti-SARS-CoV-2 IgG antibodies and the study variables were estimated using univariate analyses and chi-square test or G test. A *P*-value less than or equal to 0.05 was considered statistically significant. Uni- and multivariate logistic regression analyses were performed to explore the associations between risk factors (social, economic, and behavioral characteristics and symptoms) and the presence of anti-SARS-CoV-2 antibodies as well as the relationships between symptomatic and asymptomatic disease and the presence of these antibodies. To classify symptomatic individuals, the definitions established by the Brazilian Ministry of Health (Brasil, 2020) were used. Cases that did not meet these criteria or individuals who did not present any symptoms were classified as asymptomatic. The graphs presented herein were created in GraphPad Prism 8.0 and R Studio version 4.1.1.

## Results

### Seroprevalence

After the first wave of COVID-19, 736 individuals were invited and agreed to participate in the study. According to the serology results, 275 (37.3%) individuals were classified as reagent with regard to anti-SARS-CoV-2 IgG antibodies, 429 (58.2%) were classified as non-reagent, and 32 (4.3%) had indeterminate results. After the second wave, 601 individuals participated in the study; 284 (47.2%) were classified as reagent regarding IgG anti-SARS-CoV-2 antibodies, 274 (45.6%) were classified as non-reagent, and 43 (7.2%) had indeterminate results.

In total, only 704 individuals (95.6%) after the first wave and 558 (92.8%) after the second wave with confirmed laboratory diagnoses were selected for statistical analysis because they had accurate confirmation of the diagnosis (reagents or non-reagents). Individuals with indeterminate results, that is, with inconclusive laboratory results, were excluded from the statistical analyzes. In both periods, the seroprevalence of anti-SARS-CoV-2 IgG antibodies was higher in female individuals (first wave: *F* = 65.1%/second wave: *F* = 64.4%; *p* = 0.8009) than in male individuals (**Table 1**).

**Table 1:** Socioeconomic characteristics, prevalence of comorbidities, and risk of SARS-CoV-2 infection.

Variables	First Wave (%)	95% IC	Second wave (%)	95% IC	Univariate analysisOR (95% IC)	*P*	Multivariate analysisOR (95% IC)	*P*
Total	275 (39.1)	–	284 (50.1)	–	–			
Sex								
Female	179 (65.1)	59–70	183 (64.4)	58–70	(Ref)			
Male	96 (34.9)	29–40	101 (35.6)	30–41	0.96 (0.67–1.35)	0.8009		
Age								
≤18	18 (26.9)	3–9	20 (50.0)	4–10	(Ref)		(Ref)	
19–29	55 (47.4)	15–24	86 (96.6)	24–35	0.72 (0.35–1.48)	0.3784		
30–39	29 (31.9)	6–14	58 (33.7)	15–25	0.55 (0.2–1.20)	0.1383		
40–49	48 (38.4)	13–21	44 (42.7)	11–19	1.21 (0.5–2.58)	0.6183		
50–59	55 (44.7)	15–24	45 (60.8)	16–20	1.38 (0.65–2.92)	0.3958		
60–69	37 (37.0)	9–17	10 (21.7)	1–5	4.11 (1.50–10.5)	**0.0033**	6.41 (2.12–19.3)	**0.0009**
≥70	30 (47.6)	7–14	9 (75.0)	1–5	3.58 (1.34–9.56)	**0.0109**	3.68 (1.26–10.6)	**0.0166**
NI	3 (15.8)	–	12 (54.5)	–				
Education								
Illiterate	–	–	02 (0.70)	–	–	*		
Elementary school	38 (13.8)	9–17	23 (8.10)	4–11	4.80 (2.53–9.11)	**<0.0001**	3.55 (1.56–8.03)	**<0.0001**
High school	110 (40.0)	34–45	68 (23.9)	19–28	4.58 (2.82–7.44)	**<0.0001**	3.44 (1.98–5.93)	**<0.0001**
Undergraduate school	89 (32.4)	36–37	102 (35.9)	30–41	3.04 (1.87–4.91)	**<0.0001**		
Graduate school	38 (13.8)	9–17	84 (29.6)	24–34	(Ref)		(Ref)	
NI	0	–	05 (1.76)	–	–			
Skin color								
Yellow	3 (1.1)	–	07 (2.46)	0–4	0.40 (0.10–1.65)	0.2089		
White	62 (22.5)	17–27	60 (21.1)	16–25	(Ref)			
Black	36 (13.1)	9–17	58 (20.4)	15–25	0.59 (0.34–1.02)	**0.0590**		
Brown	173 (62.1)	57–68	153 (53.9)	48–59	0.70 (1.62–0.74)	0.7473		
NI	1 (0.4)	–	06 (2.11)	–				
Income								
≤ 1 or 2	135 (49.1)	43–55	120 (42.3)	36–48	1.22 (0.82–1.80)	0.3216		
3 or 4	53 (19.3)	14–23	66 (23.2)	18–28	0.86 (0.53–1.39)	0.5490		
≥5	79 (28.7)	23–34	84 (29.6)	24–34	(Ref)			
NI	8 (2.9)	–	14 (4.93)	–				
Comorbidity								
Diabetes	22 (8.0)	4–11	20 (7.04)	4–10	1.15 (0.61–2.15)	0.6678		
Asthma	25 (9.1)	5–12	23 (8.10)	4–11	1.13 (0.62–2.05)	0.6756		
Autoimmune disease	5 (1.8)	–	05 (1.76)	–	1.03 (0.29–3.60)	0.9590		
Cardiovascular disease	11 (4.0)	1–6	07 (2.46)	0–4	1.81 (0.70–4.65)	0.2215		
Hypertension	68 (24.7)	19–29	32 (11.3)	7–14	2.64 (1.66–4.16)	**<0.0001**		
Obesity	13 (4.7)	2–7	10 (3.52)	1–5	1.47 (0.64–3.87)	0.3626		
Tuberculosis	1 (0.4)	–	0 (0.0)	–	–			
None	157 (57.1)	–	176 (62.0)	–	(Ref)			

OR, odds ratio; NI, not informed; *, not significant. Bold means the values are statiscally significant.

**Figure 1:**
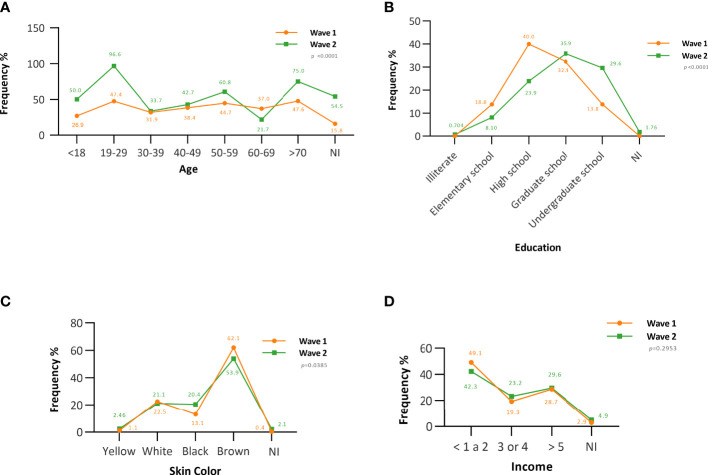
The frequency of anti-SARS-CoV-2 IgG antibodies according to socioeconomic characteristics. **(A)** Age-associated seroprevalence. **(B)** Seroprevalence associated with education level. **(C)** Seroprevalence associated with skin color. **(D)** Seroprevalence associated with family income. NI, not informed.

In the first wave, the seroprevalence was highest in individuals with advanced age [≥70 years (47.6%)], which was different from the second wave, in which younger individuals were the most affected (19–29 years: 96.6%) (**Figure 1A**). Individuals with primary and secondary education levels had the highest seroprevalence rates in the first wave, while those with higher education (35.9%) levels were more affected in the second wave (**Figure 1B**). Self-declared brown skin color (first wave = 62.0%/second wave = 53.9%; *p* = 0.0385) (**Figure 1C**) and a family income ≤2 times the minimum wage (first wave = 41.9%/second wave = 42.3%; *p* = 0.2953) (**Figure 1D**) were associated with the highest antibody rates in the two periods. The most frequent comorbidities in both periods were hypertension (first wave = 24.7%/second wave = 11.3%; *p* < 0.0001) and asthma (first wave = 9.1%/second wave = 8.10%; *p* = 0.6756). There was no correlation between seropositivity for anti-SARS-CoV-2 IgG antibody and the presence of comorbidities (**Table 1**).

### Seroprevalence associated with behavioral characteristics

**Figure 2:**
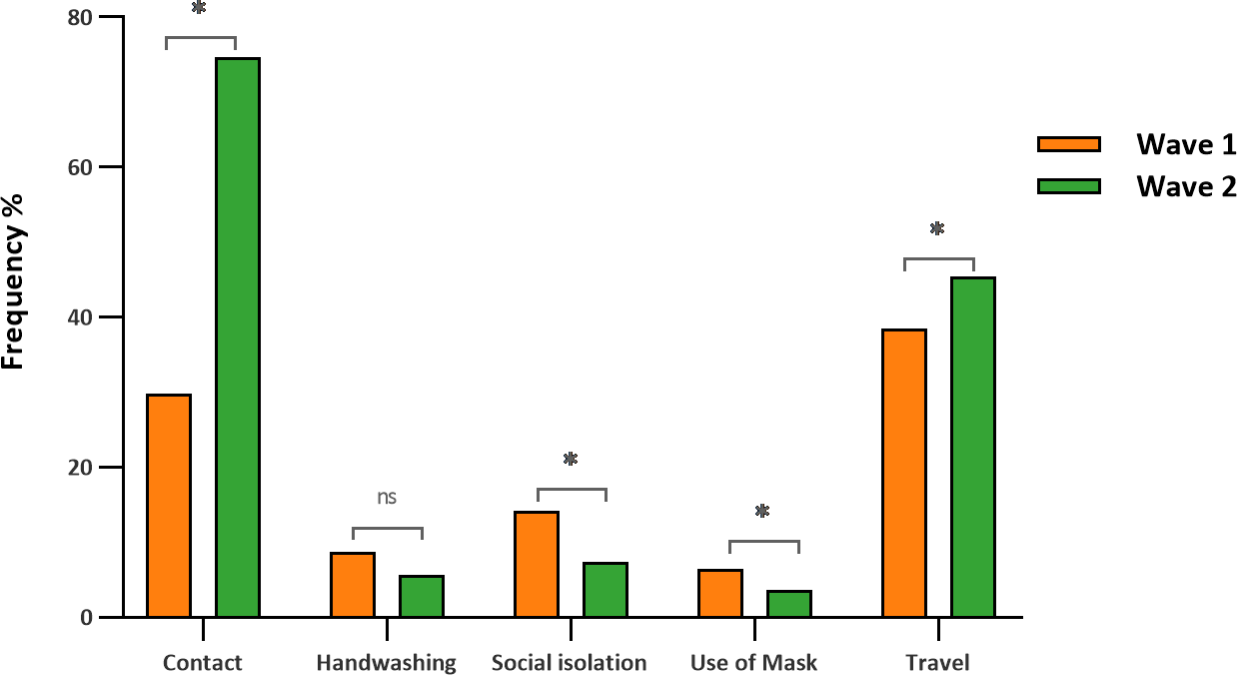
Behavioral characteristics associated with seropositivity after the first and second waves of COVID-19. *, significant values (*p* < 0.05); ns, nonsignificant values (*p* > 0.05).

During the second wave, there were increases in the number of people with a travel history (OR = 0.69; 95% CI = 0.49–0.9; *p* = 0.0346) and with contact with infected people (OR = 0.65; 95% CI = 0.44–0.95; *p* = 0.0290), resulting in higher seroprevalence compared to that in the first wave (**Table 2**). In addition, in the same period, we observed reductions in the number of people who reported wearing masks (OR = 2.89; 95% CI = 1.12–7.40; *p* = 0.0270), washing their hands (OR = 1.15; 95% CI = 0.78–2.91; *p* = 0.2169), and practicing social isolation (OR = 1.99; 95% CI = 1.13–3.48; *p* = 0.0157) (**Figure 2**).

**Table 2:** Seropositivity associated with behavioral characteristics after the first and second waves of COVID-19.

Variables	First wave (%)	95% IC	Second wave (%)	95% IC	Univariate analysisOR (95% IC)	*P*	Multivariate analysisOR (95% IC)	*P*
**Total**	275 (39.1)		284 (50.1)					
**Contact with an infected person**								
Yes	82 (29.8)	24–35	212 (74.6)	69–79	0.65 (0.44–0.95)	**0.0290**		
No	189 (68.7)	36–74	61 (21.5)	16–26	(Ref)			
NI	4 (1.5)		11 (3.87)					
**Social isolation**								
Yes	234 (85.1)	80–89	251 (88.4)	84–92	(Ref)		(Ref)	
No	39 (14.2)	10–18	21 (7.39)	4–10	1.99 (1.13–3.48)	**0.0157**	2.12 (1.13–3.94)	**0.0184**
NI	2 9 (0.7)		12 (4.23)					
**Handwashing**								
Rarely	24 (8.7)	5–12	16 (5.63)	3–8	1.15 (0.78–2.91)	0.2169		
Many times a day	250 (90.9)	87–94	252 (88.7)	85–92	(Ref)			
NI	1 (0.4)		17 (5.63)					
**Use of mask**								
Sometimes	18 (6.5)	3–9	8 (3.7)	0–4	2.89 (1.12–7.40)	**0.0270**	3.38 (1.24–9.18)	**0.0168**
Always	245 (89.1)	85–92	236 (83.1)	78–87	(Ref)		(Ref)	
NI	12 (4.4)		40 (14.1)					
**Travel history**								
Yes	106 (38.5)	32–43	129 (45.4)	39–41	0.69 (0.49–0.97)	**0.0346**		
No	167 (60.7)	55–66	147 (51.8)	45–57	(Ref)			
NI	2 (0.8)		8 (2.8)					

OR, odds ratio; NI, not informed. Bold means the values are statiscally significant.

### Risk factors associated with SARS-CoV-2 infection

**Figure 3:**
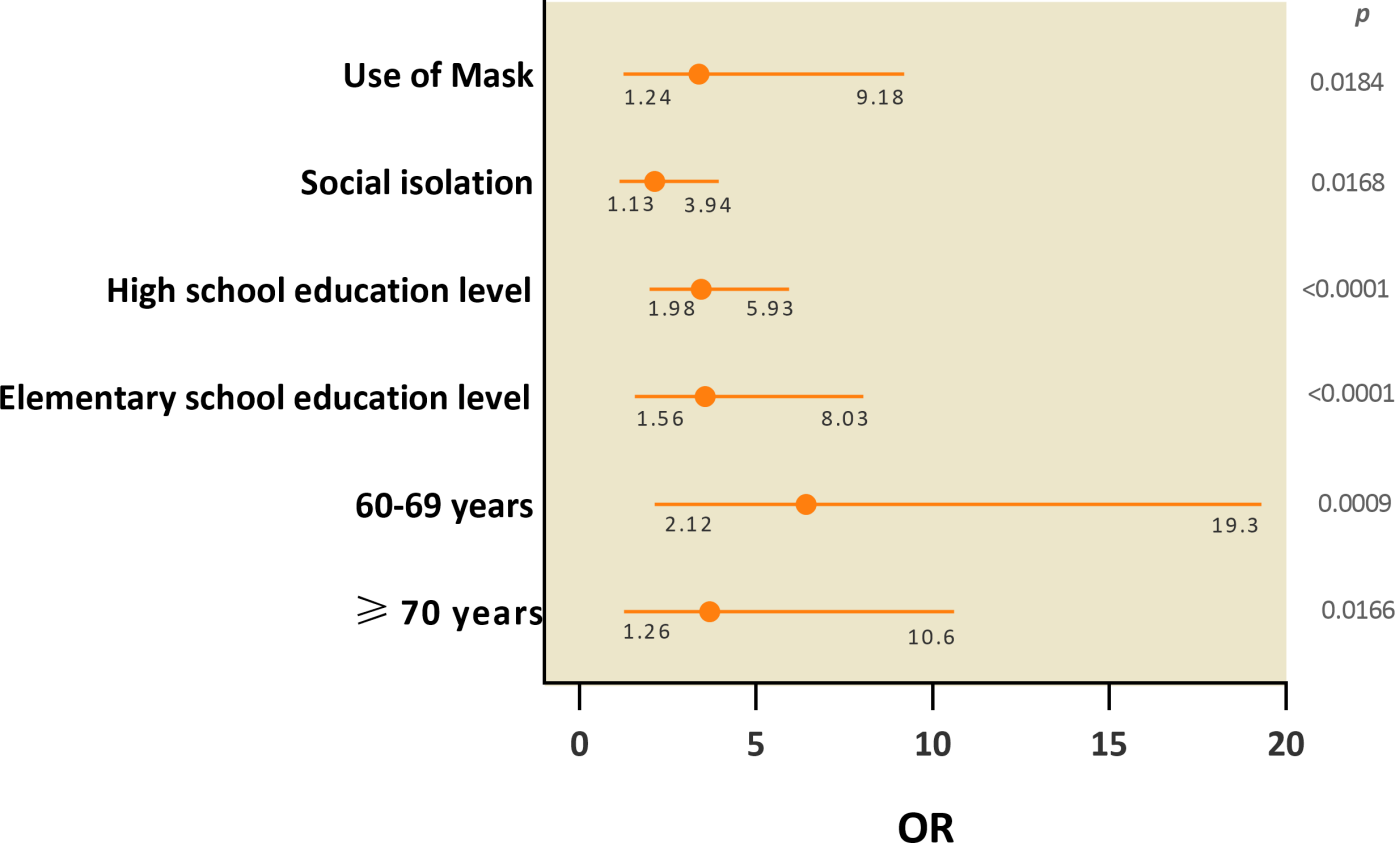
Socioeconomic and behavioral variables associated with the risk of SARS-CoV-2 infection after the first two waves of COVID-19 in the city of Belém.

Individuals aged between 60 and 69 years (OR = 6.41; 95% CI = 2.12–19.3; *p* = 0.0009) and ≥70 years (OR = 3.41; 95% CI = 1.26–10.6; *p* = 0.0166), with an elementary school (OR = 3.55; 95% CI = 1.56–10.6; *p* < 0.0001) or high school education level (OR = 3.44; 95% CI = 1.98–5.93; *p* < 0.0001), who did not use a mask frequently (OR = 3.38; 95% CI = 1.24–9.18; *p* = 0.0168), and who did not practice social isolation (OR = 2.12; 95% CI = 1.13–3.94; *p* = 0.0184) had a higher risk of SARS-CoV-2 infection in the first wave of the disease than in the second (**Figure 3**).

### Prevalence of symptoms after the first and second waves of COVID-19

**Figure 4:**
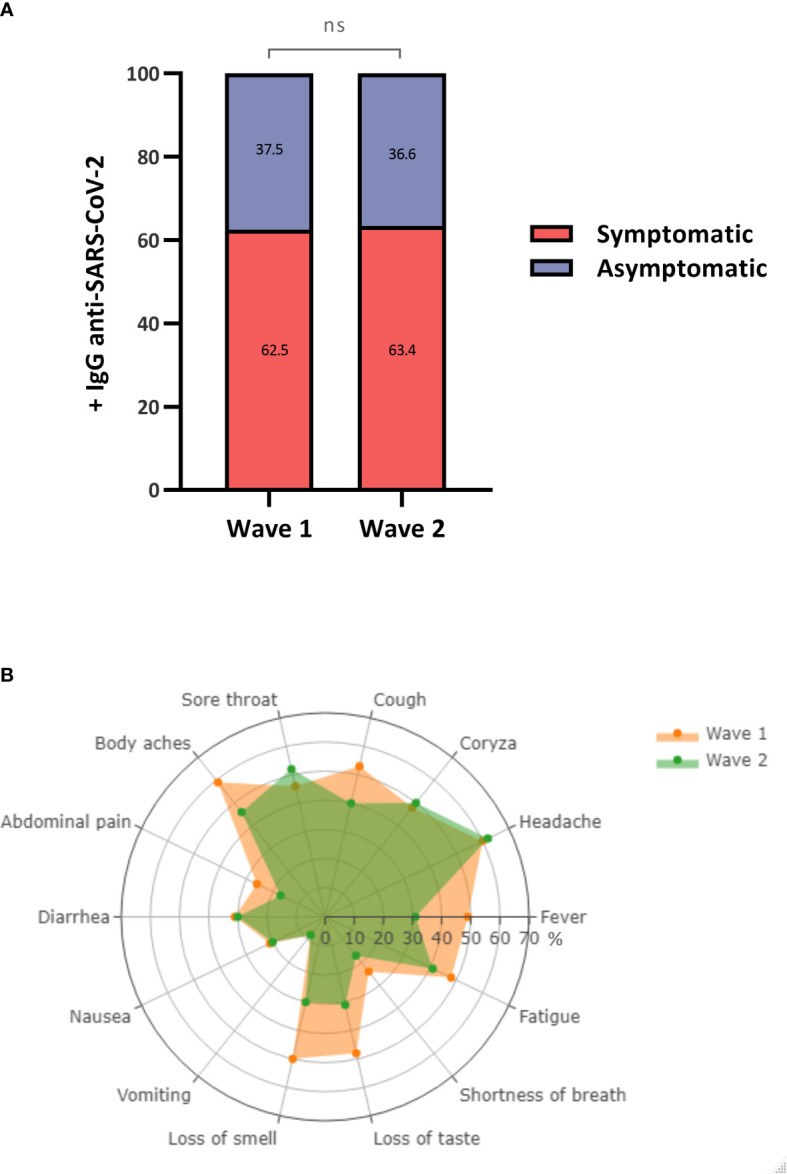
Prevalence and frequency of antibodies and symptoms among symptomatic and asymptomatic individuals. **(A)** Percentage of +/- anti-SARS-CoV-2 IgG antibodies between symptomatic and asymptomatic individuals. **(B)** Frequency of symptoms characteristic of COVID-19. ns, not significant.

Among the anti-SARS-CoV-2 IgG seropositive participants, 62.5 and 63.4% were symptomatic and 37.5 and 36.6% were asymptomatic during the first and second waves, respectively. There was no significant difference between the groups regarding seropositivity (**Figure 4A**). We found a substantial difference in symptoms between the two periods studied. Symptoms including fever (first wave = 48.7%/second wave = 30.6%; *p* = 0.0039), abdominal pain (first wave = 26.5%/second wave = 16.9%; *p* = 0.0339), loss of smell (first wave = 50.5%/second wave = 29.9%; *p* = 0.0021), and loss of taste (first wave = 48.4%/second wave = 31.3%; *p* = 0.0100) were more prevalent in the first wave than in the second wave (**Figure 4B**; **Table 3**).

**Table 3:** Prevalence of symptom characteristics of COVID-19 in IgG+ individuals.

Symptoms	First wave (%)	95% IC	Second wave 2 (%)	95% IC	*p*
Total	275 (31.9)	–	284 (50.1)	–	–
Fever	134 (48.7)	42–54	87 (30.6)	25–36	**0.0039**
Headache	164 (60.0)	53–65	176 (61.9)	56–67	0.8334
Coryza	130 (47.6)	41–53	143 (50.3)	44–56	0.7240
Cough	145 (53.5)	46–58	114 (40.1)	34–45	0.0833
Sore throat	125 (46.2)	39–51	148 (52.1)	46–57	0.9842
Body aches	161 (58.9)	52–64	132 (46.4)	40–52	0.1276
Abdominal pain	73 (26.5)	21–31	48 (16.9)	12–21	**0.0339**
Diarrhea	86 (31.3)	26–36	85 (29.9)	24–36	0.8699
Nausea	59 (21.5)	16–26	57 (20.0)	15–24	0.8222
Vomiting	23 (8.0)	0–4	24 (8.4)	5–11	0.9062
Loss of smell	137 (50.5)	43–55	85 (29.9)	24–35	**0.0021**
Loss of taste	132 (48.4)	42–53	89 (31.3)	25–36	**0.0100**
Shortness of breath	65 (24.0)	18–28	48 (16.9)	12–21	0.1306
Fatigue	130 (48.0)	41–53	118 (41.5)	35–47	0.4419

Bold means the values are statiscally significant.

## Discussion

In the present study, we performed a comparative analysis of the prevalence of anti-SARS-CoV-2 IgG antibodies against the S1 subunit of the spike glycoprotein and the factors potentially associated with infection after the first and second waves of COVID-19 in the city of Belém, the capital of the state of Pará, the largest metropolis in the Brazilian Amazon. To date, this is one of the first seroepidemiological and comparative studies that highlight changes in the affected population profile between two major periods of the pandemic. Our findings indicate an increase in seroprevalence in individuals between the first and second waves of COVID-19 in the city of Belém; moreover, the seroprevalence was higher than that observed in Manaus (first and second waves = 6.61%/first wave = 27.7%/second wave = 34.3%), which is considered one of the regions most affected by the pandemic in Brazil (Lalwani et al., 2021).

Seropositivity in young adults aged 19 to 29 years was higher in the second wave than in the first wave, during which elderly individuals had the highest rate of infection. This result is not surprising, as vaccination in individuals over 70 years of age soon after the first viral wave provided protection in this group during the second wave. Age disparities between the two outbreak periods, similar to that observed in our study, were also observed in other parts of the world (Capai et al., 2021; Visscher et al., 2022). Notably, during the second wave, high seroprevalence rates were observed in individuals with higher education levels and with high family incomes; this was most likely attributed to the gradual opening of institutions during the second period (schools, bars, workplaces, and restaurants).

Our analyses showed that behavioral characteristics, including the frequency of travel, the frequency of mask use, hand hygiene, social isolation, and contact with infected people, were related to a higher seroprevalence (Leung et al., 2020; Howarda et al., 2021). It is likely that the relaxation of prevention measures, “pandemic fatigue,” and the increasing availability of diagnostic tests influenced this pattern.

Individuals aged 60 years or older, those had been in elementary or high school education level, those who rarely used protective face masks, and those who did not practice social isolation had a higher risk of infection during the first wave of the disease than their counterparts. We attribute this increased risk to (i) the lack of vaccination of elderly individuals at the beginning of the pandemic, (ii) socioeconomic discrepancies, which are most evident in the population with low education and/or income, among the most vulnerable populations due to the lack of social isolation as a result of the need to guarantee their subsistence, and (iii) the lack of information about the proper use of masks.

The proportion of asymptomatic individuals in the seropositive population was relatively high during both periods (first wave = 37.5%/second wave = 36.6%) and was proportional to those described in other regions of Brazil (Silva et al., 2020; Borges et al., 2020; Vale et al., 2022). This finding highlights the importance of continuous monitoring and disease prevention measures, as the identification of asymptomatic individuals is still one of the main challenges and these individuals are an important source of transmission of the virus. Among the symptomatic population, symptoms such as fever, cough, abdominal pain, and loss of smell and taste were the most frequent in the first wave of the pandemic. Changes in the most frequent symptoms occurred with the circulation of different viral variants at different times; these variants showed differences in not only their genomic characteristics but also their transmissibility and pathogenesis. During the first wave, the variants circulating in Brazil were Gamma and Alpha, and during the second wave, the Delta variant predominated (Brasil, 2022b).

The main limitations of the present study, which could bias the results, were as follows: (i) the inability to investigate acute infections at the time of sample collection and to determine the circulating molecular variant, (ii) grouping of individuals based on self-reported skin color, and (iii) random sampling based on the voluntary adherence of participants. In addition, we found it difficult to obtain additional samples due to government lockdown decrees. On the other hand, our analyses provide detailed data on the socioeconomic and behavioral characteristics of the infected population (including asymptomatic and recovered patients); these data provide more accurate estimates of the prevalence of SARS-CoV-2 infection in the population of Belém in the Brazilian Amazon during the two main periods of the pandemic. This is the first epidemiological study to evaluate and compare the risk factors for SARS-CoV-2 infection shortly after the first two waves of COVID-19 in the capital of the state of Pará and to show that the pandemic waves had distinct epidemiological characteristics that need to be understood to guide the formulation of public policies that will help prevent the further spread of the infection.

In conclusion, our results showed high seroprevalence rates after the two initial periods of the pandemic in the city of Belém. These findings highlight the importance of serosurveillance, especially after the easing of prevention measures, to estimate the real impact of the COVID-19 pandemic and identify vulnerable populations and ongoing transmission. This information can guide the planning of adequate public health measures and nonpharmacological interventions in areas with few financial resources and high viral transmission rates.

## Data availability statement

The raw data supporting the conclusions of this article will be made available by the authors, without undue reservation.

## Ethics statement

This study was reviewed and approved by Ethics Committee in Research with Human Beings of the Institute of Health Sciences of the Federal University of Pará (CAAE: 31800720.1.0000.0018). The patients/participants provided their written informed consent to participate in this study.

## Author contributions

AV and MT conceptualized the study and wrote the article. Sample collection and experimental analyses were performed by MT, FL, OO, CL, AL, WB, JSG, VF, BS, BB, AC, LM and RF. The epidemiological database was compiled by RS and JLG. The statistical analyses were performed by SL and MT. Study coordination was performed by IV and AV. All authors contributed to the article and approved the submitted version.

## Funding

The study received financial support from the National Council for Scientific and Technological Development (CNPq, #302935/2021-5, and #401235/2020-3), Fundação Amazônia Pará de Amparo à Pesquisa (FAPESPA—005/2020), Coordination of Improvement of Higher Education Personnel (CAPES), and the Dean of Research and Graduate Studies at the Federal University of Pará (PROPESP/UFPA).

## Acknowledgment

The authors thank all patients who agreed to voluntarily participate in this study.

## Conflict of interest

The authors declare that the research was conducted in the absence of any commercial or financial relationships that could be construed as a potential conflict of interest.

## Publisher’s note

All claims expressed in this article are solely those of the authors and do not necessarily represent those of their affiliated organizations, or those of the publisher, the editors and the reviewers. Any product that may be evaluated in this article, or claim that may be made by its manufacturer, is not guaranteed or endorsed by the publisher.
